# HIV-1 subtype C transmitted founders modulate dendritic cell inflammatory responses

**DOI:** 10.1186/s12977-020-00526-0

**Published:** 2020-07-02

**Authors:** Evelyn Ngwa Lumngwena, Simon Metenou, Lindi Masson, Claudia Cicala, James Arthos, Zenda Woodman

**Affiliations:** 1 Division of Cardiology, Department of Medicine, Faculty of Health Sciences, University of Cape Town, Cape Town, South Africa; 2 Institute of Infectious Diseases and Molecular Medicine (IDM), University of Cape Town, Cape Town, South Africa; 3 Centre for the Study of Emerging and Re-emerging Infections (CREMER) and Virology Laboratory, Institute for Medical Research and Medicinal Plant Studies (IMPM), Ministry of Scientific Research and Innovation (MINRESI), Yaounde, Cameroon; 4 National Institute of Allergy and Infectious Diseases (NIAID), NIH, Bethesda, MD USA; 5 Disease Elimination Program, Life Sciences Discipline, Burnet Institute, Melbourne, Australia; 6 Department of Integrative Biomedical Sciences (IBMS), Faculty of Health Sciences, University of Cape Town, Cape Town, South Africa

**Keywords:** HIV-subtype C Env, Transmitter/Founder Envelopes, Inflammatory responses, Virus survival, Immunosuppression, Transmission

## Abstract

**Background:**

Heterosexual transmission remains the main route of HIV-1 transmission and female genital tract (FGT) inflammation increases the risk of infection. However, the mechanism(s) by which inflammation facilitates infection is not fully understood. In rhesus macaques challenged with simian immunodeficiency virus, dendritic cell (DC) mediated recruitment of CD4+ T cells to the FGT was critical for infection. The aim of this study was to delineate the mechanisms underlying DC-mediated HIV infection by comparing chemokine and pro-inflammatory cytokine production in response to transmitted founder (TF) and chronic infection (CI) Envelope (Env) pseudotyped viruses (PSV).

**Results:**

Monocyte-derived DCs (MDDCs) were stimulated with PSV and recombinant gp140 representing matched TF and CI pairs of four individuals and cytokine secretion measured by multiplex immuno-assay. We found that 4/9 Env induced robust MDDC inflammatory responses and of those, three were cloned from TFs. Overall, TF Env induced MDDCs from healthy donors to secrete higher concentrations of inflammatory cytokines and chemokines than those from CI, suggesting TF Env were better inducers of inflammation. Assessing the signalling pathway associated with inflammatory cytokines, we found that PSV of matched TF and CI variants and a gp140 clone activated ERK and JNK to similar levels. Recombinant soluble DC-SIGN inhibited cytokine release and activation of ERK by PSV, suggesting that Env-DC-SIGN binding was partly involved in MDDC stimulation. Therefore, Env clones might differentially stimulate MDDC immune responses via alternative, yet unidentified signalling pathways.

**Conclusion:**

Overall, this could suggest that the genetics of the virus itself influences inflammatory responses during HIV infection. In the absence of pre-existing infections, induction of greater inflammatory response by TFs might favour virus survival within the healthy FGT by driving an influx of target cells to sites of infection while suppressing immune responses via IL-10.

## Background

Inflammation of the female genital tract (FGT) facilitates HIV-1 transmission [1–3]. This could be because of mechanical damage to the protective mucosal barrier or the influx of T cells, macrophages and dendritic cells (DCs) to the FGT mucosa [2, 4]. Recruitment of HIV-permissive cells to the FGT might allow for rapid infection, transport to lymph nodes and unchecked viral replication. Haaland et al. [2] found that infection by multiple variants was associated with FGT inflammation. However, in the vast majority of cases, productive clinical infection is due to a single variant, the transmitted founder (TF) despite the presence of multiple variants in the donor [5, 6]. These findings suggest that not all variants are able to overcome an intact mucosal barrier and it has long been hypothesised that TF Envelope (Env) might provide a selective advantage during transmission. A number of studies [7, 8] to identify the elusive transmission motif have thus far suggested that only R5 tropism and potentially some Env N-glycosylation might consistently be associated with TFs [9–12]. The link between FGT inflammation, infection by TFs in the presence of an intact mucosal barrier and Env structure and function has yet to be elucidated.

In a previous study, when rhesus macaques were inoculated with simian immunodeficiency virus (SIV), DCs secreted inflammatory cytokines and chemokines (IFN-γ MIP-1α, MIP-1β, and MIP-3 α,) that recruited CCR5+ target cells to the local mucosa leading to enhanced infection [13]. Moreover, when DC inflammatory immune responses (IR) were inhibited using anti-inflammatory glycerol monolaurate, animals were protected against SIV infection [13]. This suggested that similar to other pathogens, HIV-1 deregulation of DC cytokine secretion might play an important role in transmission [14, 15].

It was also found that when HIV-1 Env binds to DC-SIGN it triggers *trans*-infection of CD4+ T cells, deregulation of DC function, prevention of DC maturation and inhibition of IL-12 expression [16–18]. In addition, when monocyte derived DCs (MDDCs) were exposed to HIV-1, IL-10 secretion [19, 20] was enhanced while the release of IL-12 and its capacity to stimulate T cell proliferation was inhibited [21]. Cohort studies showed that concentrations of pro-inflammatory cytokines, IL-8, IL-1 and TNF-α, and pleiotropic cytokines such as IL-10 and IL-6 were found to be abnormally high in plasma of HIV infected patients with a concomitant decrease in IL-2, IL-12 and IFN-γ levels [22–32]. Moreover, cervico-vaginal lavages (CVL) of HIV infected women sampled within weeks of infection had significantly elevated levels of IL-6, IL-10, and IL-12, compared to HIV negative women whereas, IL-1α, IL-1β, IL-8, IL-6, IP10 and IL-10 were increased in plasma of the same patients [22, 33]. We had previously shown that MDDCs exposed to Env of TFs secreted higher levels of IL-10 than those stimulated with Env isolated from variants during chronic stages of infection (CI) [34]. To understand the role of HIV-1 subtype C Env in local inflammatory responses that might lead to new HIV infections, we investigated whether HIV subtype C Env isolated during acute infection (AI) and CI induce distinctive inflammatory responses in healthy MDDCs.

Previous findings suggested that binding of Env to DC-SIGN triggered different signalling pathways which culminated in alternative outcomes. For instance, HIV Env binding to MDDCs via DC-SIGN led to increased Rho-GTPase activity required for the formation of virological synapses [35] whereas the expression of full-length HIV transcripts required Toll-like receptor-8 (TLR-8) [36]. Binding of gp120 to DC-SIGN led to IL-10 secretion via the activation and phosphorylation of ERK [37] and DC-SIGN-mediated modulation of TLR-dependent activation of NF-kB [38]. Shan et al. [37] suggested that virus genetics influenced whether MDDCs secreted IL-10 after exposure to gp120. Furthermore, Wilflingseder et al. [39] suggested that HIV-1 gp120 differentially activated ERK1/2 and p38 MAPK which lead to alternative DC phenotypes. Together, these findings suggested that different Env clones might differentially activate MAPKs, leading to alternative DC IR that either favour virus clearance or HIV-1 transmission. Therefore, this study hypothesised that in the absence of a pre-existing FGT inflammatory response TF Env modulates DC IR that initiate the recruitment of HIV-permissive cells, leading to productive clinical HIV infection.

## Results

### MDDC secretion of inflammatory cytokines and chemokines in response to HIV-1

**Fig. 1:**
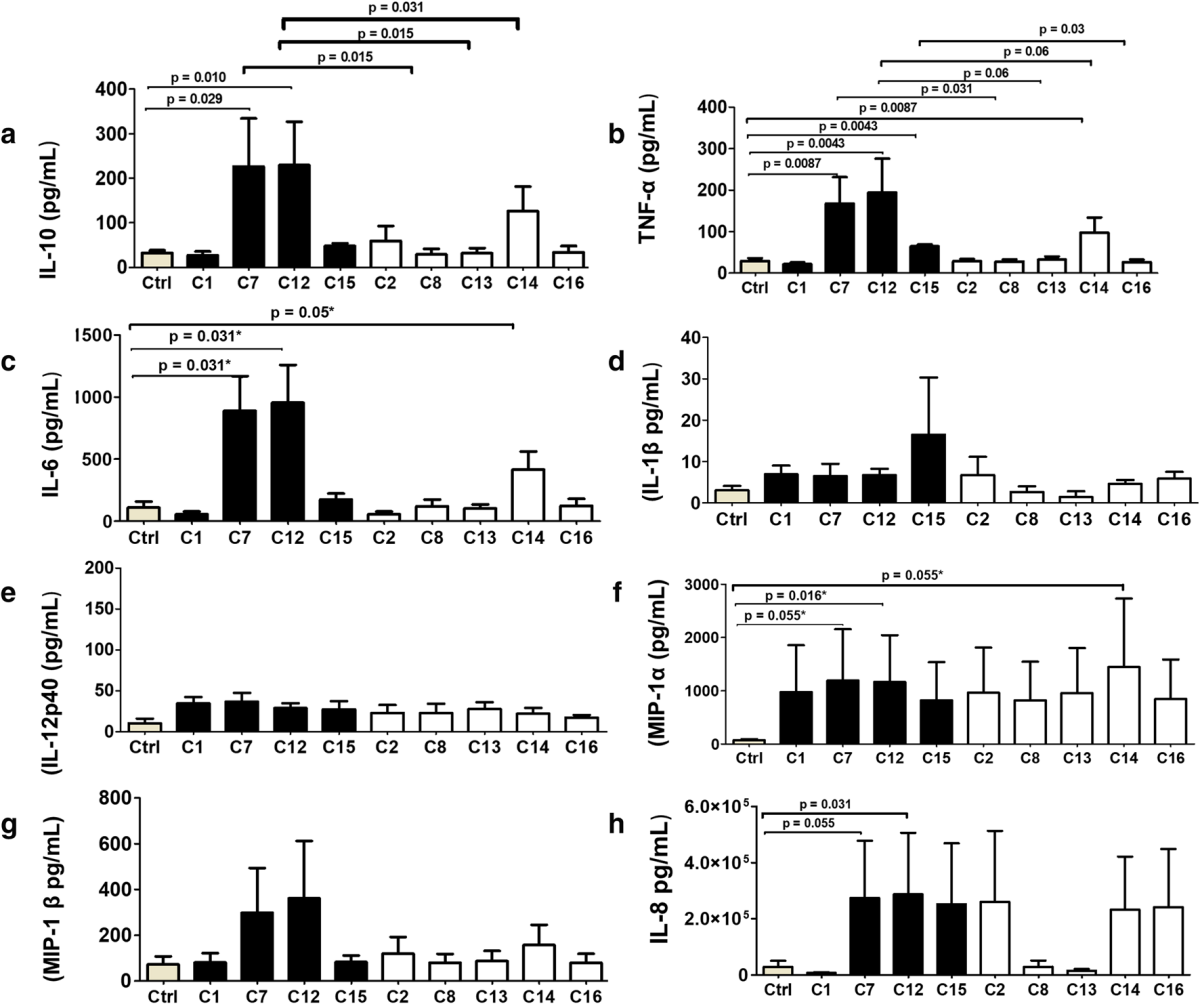
HIV-1 subtype C Envelopes induce inflammatory immune response. Monocyte derived dendritic cells (MDDC) were stimulated with pseudovirus (PSV) of Envelopes (Env) cloned from variants infecting four participants during acute (TF, black bars) and chronic (CI, white bars) stages of infection. Levels of **a** IL-10, **b** TNF-α, **c** IL-6, **d** IL-1β, **e** IL-12p40 **f** MIP-1α, **g** MIP-1β, and **h** IL-8 in the culture supernatants of MDDC were measured by Luminex assay. PSV generated using pSG3∆Env only was used as negative control (Ctrl, grey bar). Mann–Whitney test was used to compare cytokine levels to the negative control and Wilcoxon Rank Signed test was used to compare paired Env from the same participant (TF vs CI). Bar graphs represent medians of each cytokine (pg/mL) concentration of 8, 7, 6, 5 and 5 biological repeats for IL-8, IL-10, TNF-α, IL-6 and chemokines, respectively. Error bars show standard deviations (SD). P-values were adjusted for multiple comparisons by the false discovery rate step-down procedure

During an antigenic challenge, DCs secrete a combination of soluble mediators which together with co-stimulatory molecules may influence the strength and breadth of the IR [40, 41]. Therefore, in addition to the anti-inflammatory cytokine IL-10 (Fig. 1a) [34], we also determined the effect of PSV on MDDC secretion of pro-inflammatory cytokines (TNF-α, IL-6, IL-1β, IL-12p40) (Fig. 1b–e) and chemokines (MIP-1α, MIP-1β, IL-8) (Fig. 1f–h). MDDCs from healthy donors were stimulated with PSV of four TF (C1, C7, C12 and C15) and matched CI Env (C2, C8, C13, C14 and C16) and cytokines were measured by Luminex. Stimulation of IL-8 release was highly variable between donors in response to the different clones and all PSV clones seemed to be poor inducers of IL-12p40 relative to the background control. On the other hand, C7, C12, C14 and C15 were better stimulators of TNF-α, IL-6, MIP-1α, MIP-1β and IL-8 secretion than the negative control with the respective cytokine and chemokine concentrations ranging from 26–200, 55–955; 845–1446; 80–361 and 7285–287,334 pg/mL. C7 and C12 induced the secretion of significantly higher concentrations of TNF-α (p = 0.0086, p = 0.0043) IL-6 (p = 0.031, p = 0.031), MIP-1α (p = 0.055, p = 0.016) and IL-8 (p = 0.055, p = 0.031) (Fig. 1).

**Fig. 2:**
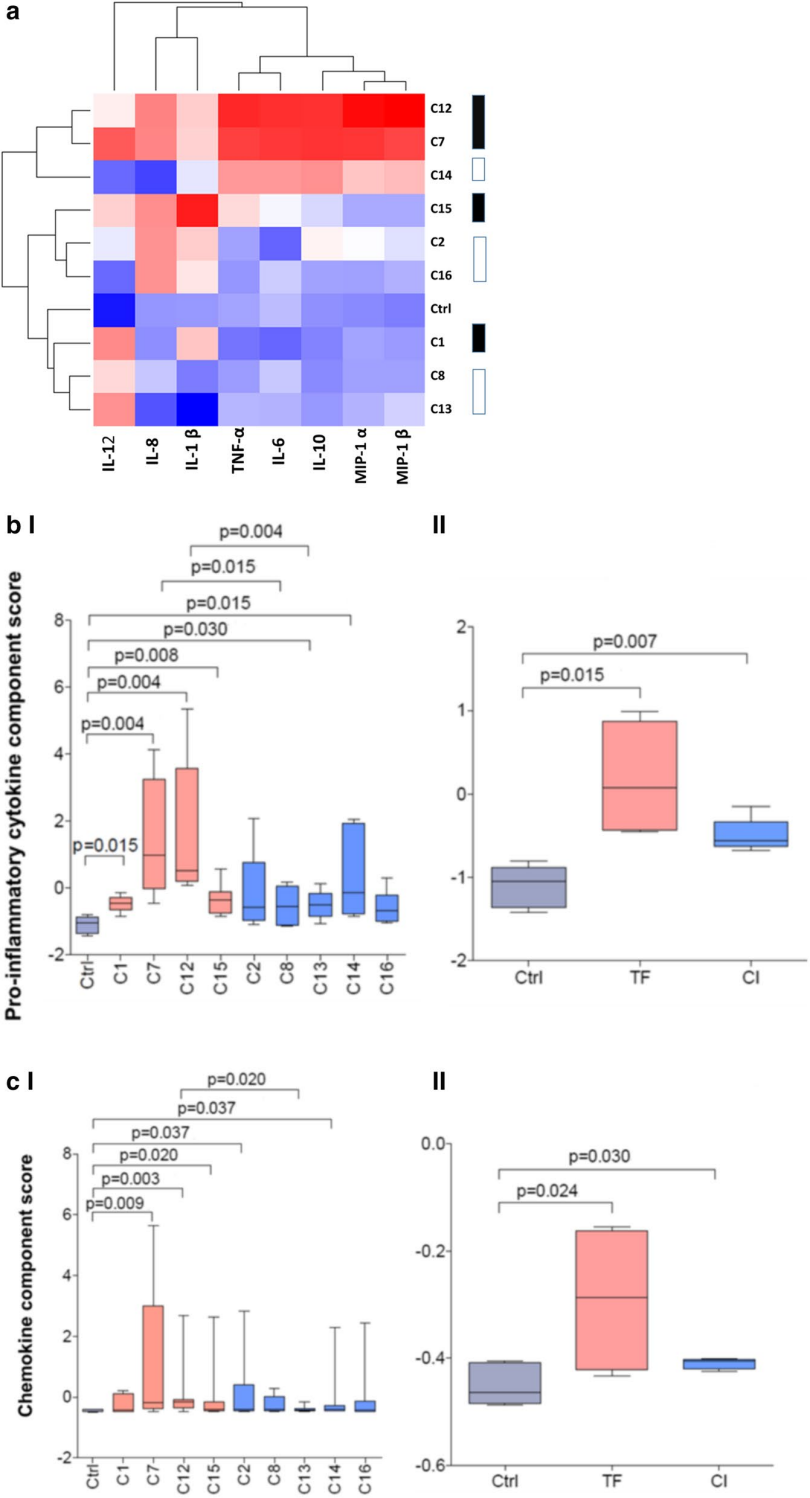
Envelope clones cluster into two groups according to inflammatory immune response. **a** Unsupervised hierarchical clustering (in R) was used to visualize all 9 differentially released cytokine concentrations secreted in response to pseudovirus (PSV) stimulation of MDDCs, and to cluster different Envelope (Env) clones according to similarity of their cytokine expression profiles irrespective of their sequence and cytokine function. Cytokine concentrations are indicated using a colour scale, ranging from blue (low), through white, to red (high). The dendrogram above the heat map illustrates degrees of relatedness between cytokines, while the dendrogram on the left illustrates Env clusters according to their capacity to induce cytokine secretion. The identity of the Env clones are indicated on the right of the heat map with transmitted founder (TF) and chronic infection (CI) clones indicated by black and white keys, respectively. Cytokines were grouped according to whether they were **b** pro-inflammatory (IL-1β, TNF-α, IL-6) or **c** chemokines (MIP-1α, MIP-1β and IL-8) and inflammatory cytokine component scores were generated using principal component analysis and plotted as bar graphs. The pro-inflammatory I) and chemotactic II) component scores of TF clones and CI clones are indicated as pink and blue bars, respectively, with boxes representing the interquartile ranges, lines within boxes represent medians and whiskers represent minimum and maximum values

To determine overall inflammatory response patterns, hierarchical clustering and exploratory factor analysis were performed of all the cytokines and chemokines induced by all PSV Env tested. Most of the cytokines clustered together in Factor 1, with pro-inflammatory cytokines (IL-6 and TNF-α), chemokines (MIP-1α and MIP-1β) and anti-inflammatory IL-10 grouping together (Fig. 2a and Table 1). IL-8 and IL-1β were closely related and grouped together in Factor 2 (Table 1). Although IL-12p40 clustered more closely with Factor 1 than Factor 2, this cytokine had the greatest degree of uniqueness (Table 1) and clustered separately in the unsupervised hierarchical exploratory analysis (Fig. 2a). Therefore, the release of IL-6, TNF-α, MIP-1α, MIP-1β and IL-10 by MDDCs in response to PSV seemed to be linked.

**Table 1:** Factor loadings and unique variances of cytokines secreted by the MDDCs in response to pseudovirus stimulation

Variable	Factor 1	Factor 2	Uniqueness
IL-10	*0.9759*	− 0.0451	0.0101
IL-6	*0.9204*	− 0.1996	0.0310
IL-1β	0.3595	*0.7651*	0.2693
TNF-α	*0.9661*	− 0.0845	0.0032
IL-12p40	*0.2434*	0.0772	0.5201
IL-8	0.4674	*0.6360*	0.3633
MIP-1α	*0.9684*	− 0.1779	0.0030
MIP-1β	*0.9732*	− 0.1125	− 0.0004

Factor loadings close to 1 indicate strong relatedness and very low values indicate uniqueness. Factor loadings greater than 0.6 (in italic) are considered for relatedness [42]

PSV clustered into two groups: those that tended to induce high levels of cytokines (upper three rows) and those that were weak stimulators of MDDC cytokine release (lower six rows) (Fig. 2a). PCA was used to generate principal component scores for each PSV, representing overall pro-inflammatory cytokine responses (TNF-α, IL-6, IL-1β) (Fig. 2b) and overall chemokine responses (MIP-1α, IL-8 and MIP-1β) (Fig. 2c). C1, C7, C12, C13, C14, and C15 had significantly higher pro-inflammatory component scores compared to the control (Fig. 2b I). Of these six clones, four represented TF variants whereas two were CI Env from the same participant. For the chemokine component scores, only C7 and C12, both TF, had significantly higher scores than controls after adjusting for multiple comparisons (Fig. 2c I).

### Transmitted founder Envelopes preferentially induced MDDC secretion of inflammatory cytokines and chemokines

Cytokine induction seemed to be associated with time post-infection as C7, C12 and to a lesser extent, C15, three of the four best inducers of cytokine release were cloned from TF variants (Fig. 1). For example, CAP206C7 and CAP210C12, induced the secretion of more TNF-α (p = 0.013) and IL-6 (p = 0.06) than their matched CI clones, CAP206C8, CAP210C13 and CAP210C14 (Fig. 1b, c). Furthermore, TF Env seemed to be better inducers of inflammation as all TF clones, C1, C7, C12, and C15 had higher pro-inflammatory component scores than the control (Fig. 2b) and TFs C7 and C12 proinflammatory scores were significantly greater than their corresponding CI (p = 0.0152 and 0.0043 respectively). Only C12 had a significantly higher chemokine score (p = 0.028) than its matched CI counterpart (Fig. 2a). In addition, only TF PSV had a tendency to induce IL-1β although this did not reach significance. When clones were grouped according to time of sampling, both the pro-inflammatory (Fig. 2b II) and chemokine (Fig. 2c II) component scores of TF and CI clones were significantly higher than the controls and TF values were greater than that of the CI clones, although the difference did not reach significance.

**Fig. 3:**
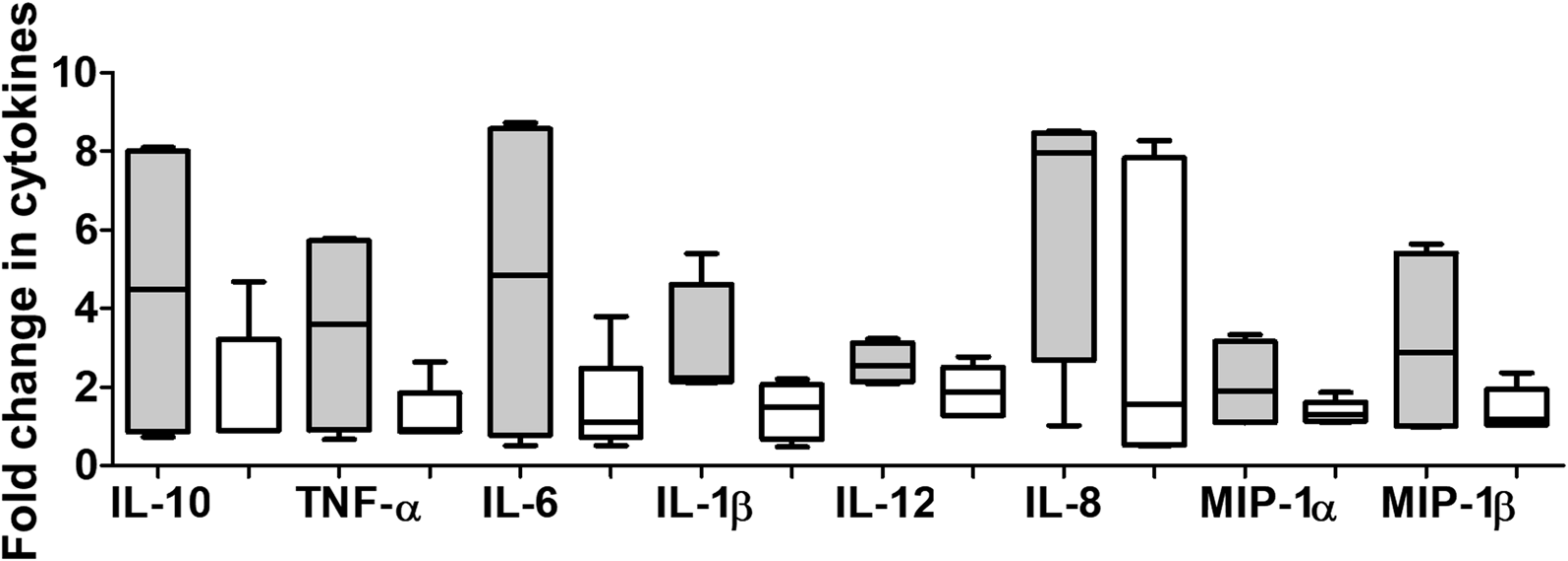
Transmitted founder Envelopes induce more inflammatory immune response. Envelope clones (Env) were grouped according to time post-infection and the cytokine concentrations (pg/ml) secreted in response to transmitted founder (TF, grey bar) and chronic infection (CI, white bar) clones were compared to background. The fold change for each cytokine and chemokine above background for four biological repeats are indicated. Mann–Whitney t-test was used to compare the median value of the fold-change of cytokine concentration of four independent MDDC donors

To circumvent experimental and donor variation between biological repeats, PSV were grouped into TF and CI variants and the concentration of each cytokine was expressed relative to the background of each biological repeat before comparing the median values. Overall, TF PSV were better at inducing each cytokine than their matched CI clone. MDDCs stimulated with TF Env tended to release two–eightfold higher concentrations of all cytokines, compared to levels induced by CI PSV that increased by only two–fourfold above background (Fig. 3).

### The role of DC-SIGN in MDDC secretion of inflammatory cytokines and chemokines

**Fig. 4:**
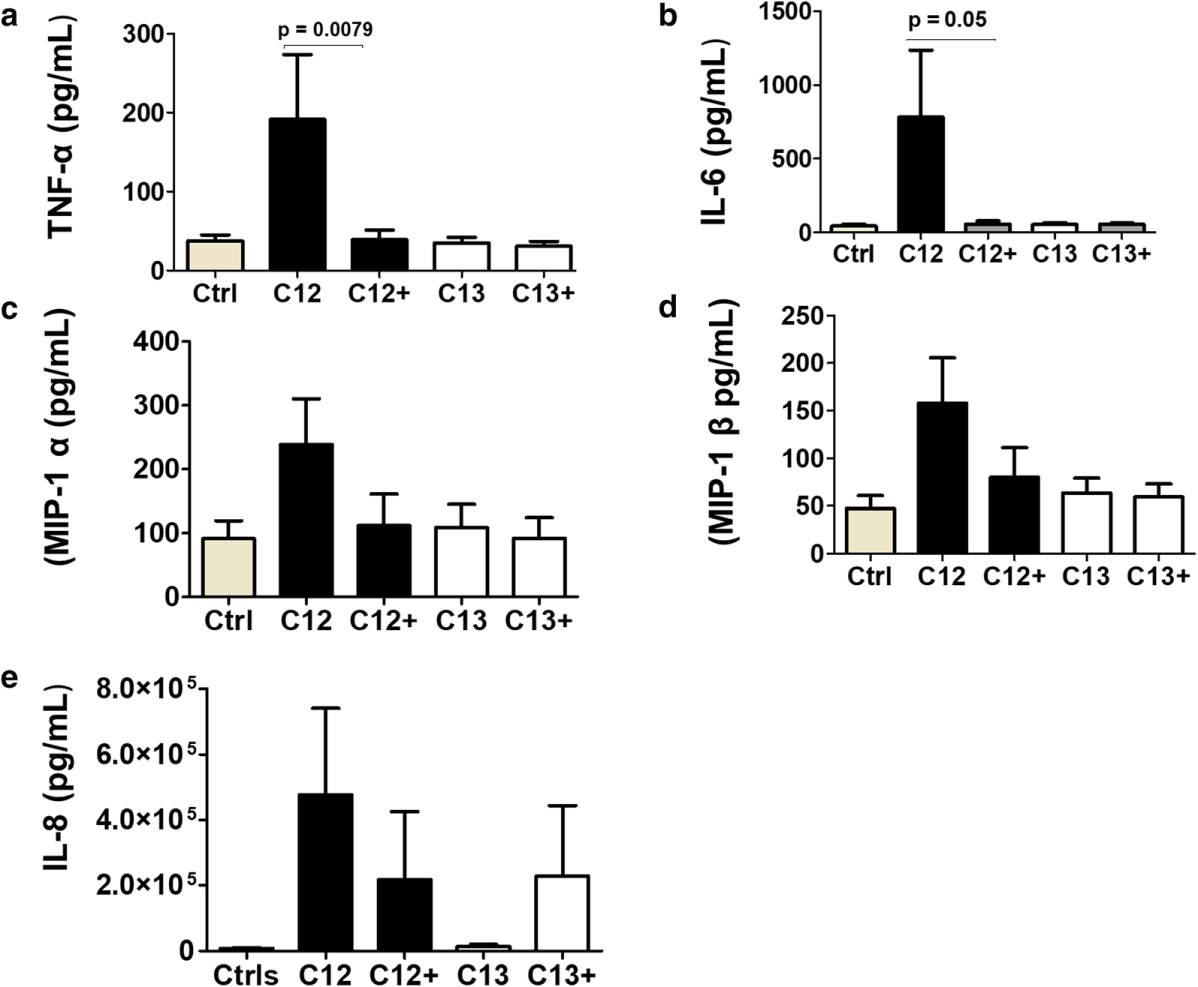
Role of DC-SIGN in inducing MDDC inflammatory response. C12 and C13 pseudovirus (PSV) were pre-incubated in the presence and absence of recombinant DC-SIGN before stimulation of MDDCs. The concentrations of secreted TNF-α, IL-6, MIP-1α, MIP-1β and IL-8 were determined by Luminex assay. Wilcoxon Rank Signed test was used to compare the cytokine levels of four independent MDDC donors and error bars indicate the standard deviation (SD)

In a previous study we stimulated MDDCs with PSV in the presence of recombinant DC-SIGN to determine whether IL-10 release was due to Env-DC-SIGN binding. DC-SIGN inhibition was shown to lower the secretion of IL-10 by MDDCs [34]. As pro-inflammatory cytokines and some chemokines grouped together with IL-10 in the hierarchical clustering, we determined whether recombinant DC-SIGN would also inhibit the release of the other inflammation modulators. Two matched clones (C12 and C13) were selected that represented the best and weakest inducers of proinflammatory cytokines and chemokines. Inhibition of C12 PSV DC-SIGN interaction with recombinant DC-SIGN significantly reduced MDDC secretion of IL-6 and TNF-α levels while inhibition of chemokine secretion did not reach statistical significance (Fig. 4). On the contrary, inhibition of MDDC cytokine and chemokine secretion in response to C13, the matched CI clone of C12, was not observed. This is likely due to C13 being a very poor inducer of all cytokines and chemokines, limiting the detection of changes in cytokine and chemokine concentrations in the presence of recombinant DC-SIGN. However, we cannot exclude that preparation of recombinant protein did not co-purify contaminants or alter the structure of soluble DC-SIGN which might have influenced its binding to Env. Despite these limitations, apparent inhibition of cytokine release in the presence of recombinant DC-SIGN suggests that DC-SIGN-Env interactions are important.

### Envelope activation of MAPK kinase signalling

**Fig. 5:**
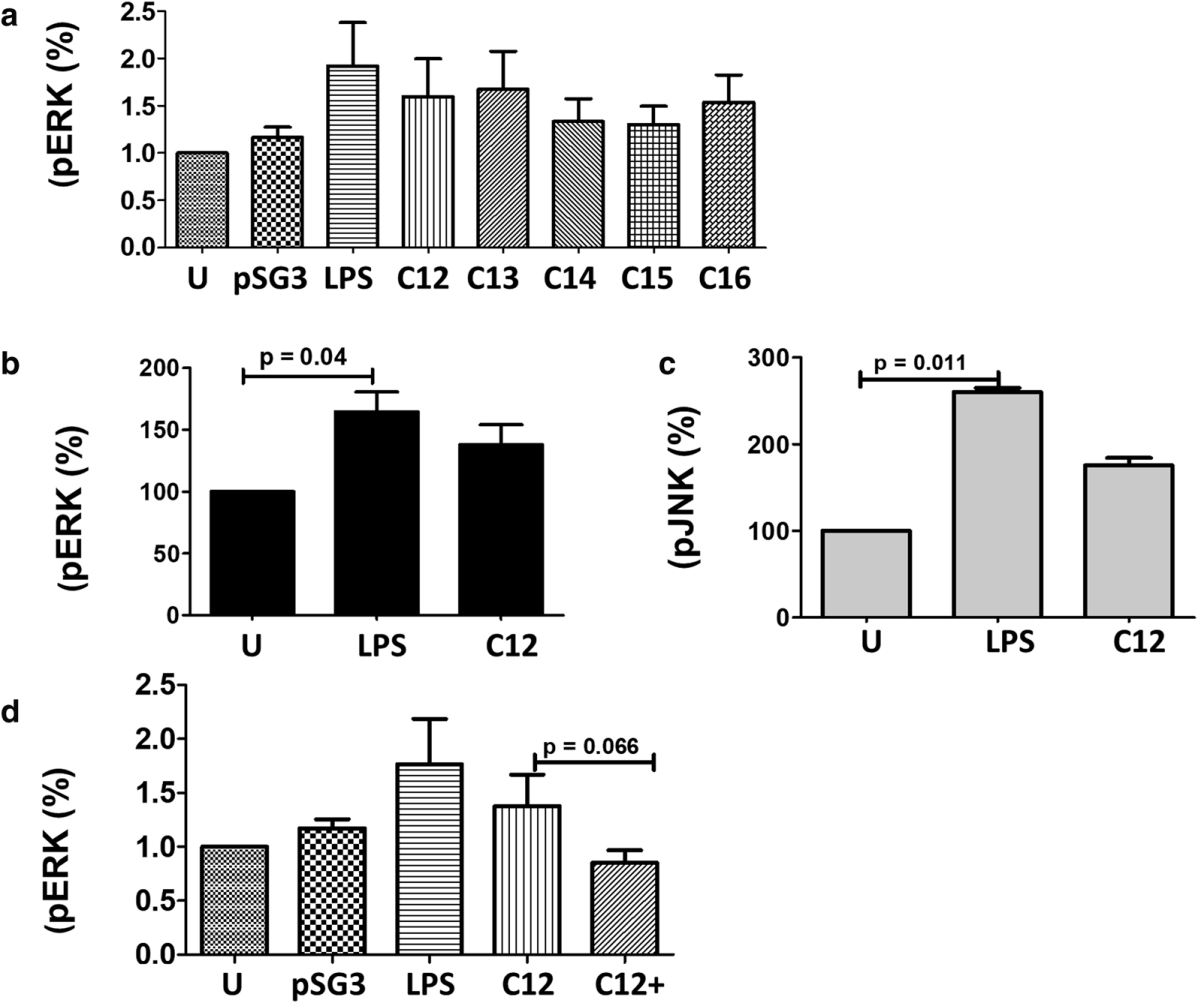
HIV-1 Envelope induces MAPK activation. A) Following stimulation of day six MDDCs with pseudovirus generated with C12, C13, C14, C15 and C16 Envelope (Env) clones, the phosphorylation of ERK was measured by Western blotting. Image J software was used to quantify band intensity and average intensity of at least four independent experiments are shown relative (%) to background signal (cells treated with pSG3ΔEnv only). Day six MDDCs were rested and stimulated with C12 (TF) gp140 and the phosphorylation of B) ERK and C) JNK was measured by flow cytometry. Flowjo software was used for analysis and mean fluorescent intensity (MFI) of two independent experiments are indicated relative (%) to background control (medium only), with error bars representing SD. D) MDDCs were stimulated with C12 PSV with (C12+) and without (C12) pre-incubation with anti-DC-SIGN monoclonal antibody and pERK levels were determined by Western blotting and expressed relative to background (%). Bar graphs indicate the mean of at least four independent experiments with error bars representing SD. A medium only (U) negative control and a lipopolysaccharide (LPS) positive control were included in each experiment. Mann–Whitney test (**a**, **d**) and one-way Anova (**b**, **c**) were used to compare means of pMAPK for both experiments

As TF and CI PSV seemed to differ in their ability to induce MDDC secretion of cytokines and chemokines, we hypothesized that differences in Env structure might activate either alternative signaling pathways or elicit a more robust response due to higher affinity binding with cell-surface receptors. Previous studies showed that gp120 protein stimulated MDDC ERK phosphorylation, impaired maturation of DCs and induced inflammation [37]. To investigate if PSV Env differentially activated MAPK signaling pathways, pERK levels were detected by WB after stimulation of MDDCs with C12, C13, C14, C15 and C16 PSVs. These clones were selected because they stimulated MDDCs to secrete different levels of cytokines and chemokines which might be reflected in changes in pERK levels. LPS was included as a positive control in this experiment because it has been shown to stimulate ERK phosphorylation [43]. PSV clones stimulated an increase in pERK levels relative to the control, although only LPS induced pERK to statistically significant levels (Fig. 5a). When we also employed flow cytometry based analysis to follow Env-mediated increased protein phosphorylation in MDDCs, LPS, C12 and C13 induced a twofold increase in phosphorylation of ERK and JNK over the control (data not shown). Therefore, although C13 PSV is a poor inducer of cytokine and chemokine secretion, it is able to stimulate both ERK and JNK phosphorylation. To confirm that Env was essential for the activation of ERK and JNK, purified gp140 was used to stimulate MDDCs. C12 gp140 stimulated MDDCs to phosphorylate ERK and JNK similar to C12 PSV although only LPS induced MDDCs to generate significantly higher levels of pERK and pJNK compared to background (Fig. 5b, c). When C12 PSV DC-SIGN interaction was inhibited by recombinant DC-SIGN, MDDC pERK levels decreased significantly (Fig. 5d), suggesting that DC-SIGN interactions in part were contributing to ERK activation, in line with our findings regarding IL-10 [34] and inflammatory cytokine secretion (Fig. 4). Therefore, the ability of C12 PSV and gp140 to induce MDDC activation of ERK and JNK may in part involve interactions between DC-SIGN and Env although we cannot rule out the influence of other DC receptors.

Therefore, Env and DC-SIGN interactions are potentially involved in both the activation of ERK and the stimulation of MDDCs to release cytokines. However, a statistically significant correlation between the activation of MAPK signaling pathways and the extent to which clones induced the secretion of cytokines and chemokines was not observed. For example, C12 and C13 PSV both induced the phosphorylation of ERK and JNK but only C12 induced significant increases in secretion of cytokine and chemokines by MDDC. It is thus possible that Env binding to DC-SIGN activates not only MAPKs but also an unidentified signaling pathway(s) linked to cytokine secretion.

## Discussion

Inflammation in the FGT is associated with increased risk of HIV infection, [44] multiple variant transmission, [2] and infection by less infectious virus [3]. However, the underlying mechanisms and causes of inflammation in a healthy genital mucosa are not fully understood. HIV may directly induce inflammatory responses needed to facilitate the establishment of infection [45] or inflammation may be pre-existing, caused by factors such as sexually transmitted infections (STIs) [2, 46] and dysbiosis [45]. In rhesus macaques, it has been shown that DCs play a critical role in the early stages of SIV infection by producing chemotactic cytokines that recruit CD4+ T cell targets for SIV infection [13]. It is thus possible that DCs play a similar role during the early stages of HIV infection. To investigate this, we compared inflammatory cytokine and chemokine production by MDDCs in response to pseudotyped Env cloned longitudinally from women infected with a single HIV variant.

IL-10, MIP-1α, MIP 1β, IL-6, and IL-8 grouped together when exploratory factor analysis was used to classify the cytokines, suggesting their functions influenced one another. It is tempting to consider that the observed DC IR might provide an advantage during transmission as three of the four clones that induced the release of high levels of cytokine were AI clones. Li et al. [3] found that a few days following macaque inoculation, pDCs just beneath the endocervix expressed high levels of MIP-1α, MIP-1β, MIP-3a, IFN-α and IFN-β and the chemokine levels were associated with enhanced SIV infection (13). Given their chemoattractant function it is possible that increased chemokine secretion helped to recruit CCR5 expressing cells to areas of infection, thereby facilitating viral replication. This was in fact seen with the influx of CD4+ cells to local infectious foci as early as 1–3 days post viral inoculation. Moreover, inhibition of inflammation by application of glycerol monolaurate significantly reduced target cells and inflammatory cytokines in the genital areas [13].

When matched pairs of Env were compared, the mean proinflammatory and chemokine scores for TFs were higher than those of CI clones. HIV-1 subtype C TF Env might have the advantage of skewing DC inflammatory responses in their favour to facilitate transmission. The in vivo “cytokine storm” associated with acute HIV-1 infection supports the suggestion that increased inflammatory responses favour viral replication most likely through the recruitment of HIV permissive cells [22, 47]. However, this might not be true for all TFs, and the absence of this advantage might have consequences later during infection. Of note, the TF of the fourth matched pair, that did not induce an inflammatory cytokine profile, was cloned from a slow progressor [48]. However, although it is tempting to speculate that TF variants have a selective advantage of inducing inflammatory responses in the FGT, our sample size is too small to reach definite conclusions.

The pathogenic products of *Mycobacterium tuberculosis* and HIV-1 (ManLAM and gp120 mannose residues, respectively) induced Raf-1 phosphorylation and activated Raf-1 promoted HIV-1 gene expression in DCs [36]. Gringhuis et al. [38, 49] found that binding of ManLAM to DC-SIGN activated Raf-1 of the Ras-Raf-MEK-MAPK pathway which resulted in NF-kB-stimulated expression of cytokine genes, including IL-10. Furthermore, Env mediated activation of ERK1/2 and p38 MAPK differed between Env clones [39] suggesting that MAPK signalling via Raf-1 might be differentially activated by HIV-1 variants. To determine whether some Env were better able to activate MAPK, with concomitant aberrant cytokine profiles, we determined whether the Env clones were able to stimulate the phosphorylation of MDDC ERK and JNK.

Following PSV and gp140 stimulation of MDDCs, we detected varied phosphorylation of the MAPKs across the clones. There was no overall significant correlation between MDDC cytokine secretion and ERK activation possibly due to the small sample size in conjunction with the high variation between MDDC donor responses. C13 consistently failed to induce MDDCs to secrete cytokines above that of background and yet this clone was able to stimulate ERK phosphorylation. Similarly, C16 PSV was also unable to stimulate MDDC inflammatory response but did increase pERK levels. This suggests that cytokine secretion in response to HIV is modulated by more than one MDDC signalling mechanism. Blocking Env-DC-SIGN interaction inhibited cytokine release and ERK phosphorylation, demonstrating that DC-SIGN is involved in triggering the inflammatory response. We cannot exclude the possibility that recombinant soluble DC-SIGN and anti-DC-SIGN antibodies non-specifically inhibited MDDC signalling pathways independent of DC-SIGN and/or that Env binds to alternative receptors. However, we have reported that PSV bound Raji cells via DC-SIGN and MDDC-associated virus *trans*-infected TZM-bl cells, confirming Env and DC-SIGN interactions [34]. Furthermore, we showed that PSV binding to Raji-DC-SIGN cells varied between clones, suggesting that differences in Env structure might influence the interaction with DC-SIGN which could activate alternative signalling molecules. Variation in Env N-glycan structures do alter binding affinity to DC-SIGN which changes its ability to interact with more than one signalling molecule [50]. However, the mechanism of how this occurs remains unknown [51].

## Conclusion

Although inflammation within the FGT is linked to HIV-1 infection [3, 52], the factors initiating the “cytokine storm” are unknown and may depend on several factors. Here we show that in the absence of pre-existing inflammation, the virus itself could modulate MDDC inflammatory responses via its Env glycoprotein interaction with DC-SIGN. Furthermore, although based on a small sample size, single variant TF PSV tended to stimulate higher levels of inflammatory cytokines and chemokines compared to CI PSVs, suggesting that DC IR could play a role in HIV transmission.

## Materials and methods

### Samples

Full-length *envelope* (*env*) from four HIV positive women (CAP45, CAP206, CAP210 and CAP239) sampled 2–5 weeks post-infection (wpi) (AI) with follow up longitudinal samples collected 2–3 years post infection (ypi) (CI) were used for this study (Table 1) (Accession numbers: JX976722.1, KC863497.1, FJ443390.1, 47:KC833437.1, KJ700457.1, DQ435683.1, KC863280.1, KC863276.1, FJ443142.1, HQ625601, HQ625600.1, FJ443357.1, HQ625599.1, KC863388.1, HQ625599.1 (www.ncbi.nlm.nih.gov). Single genome amplification (SGA) and sequencing indicated that all participants were infected with a single variant at transmission [5]. Nine functional Env clones (numbered C1, C2, C7, C8, and C12-C16), a subset of 36 generated from SGA—derived PCR products, were cloned into pcDNA/his-topo (Invitrogen) or pTarget (Promega) mammalian expression vectors according to manufacturer’s instructions. The functional clones selected in this study were sampled from four participants infected with a single variant at 2–5 wpi with follow-up 2–3 years later [5]. The CI clones (C2, C8, C13 and C14) were selected because they had N-glycosylation profiles identical to the consensus sequence of circulating variants at CI. Potential N-glycosylation (PNG) frequency analysis was carried out on 36 matched Env consensus sequences from AI and CI (accession numbers: (JX976651.1, FJ443216.1, FJ443182.1, FJ443984.1, KC894137.1, KC863412.1, JN681229.1, KC863558.1, KC863493.1, KC863519.1, KC863542.1, KC833437.1, HQ625599.1, KC863410.1, JX976681.1, HQ625602.1, KC863457.1, HQ625605.1, FJ443279.1, FJ443963.1, KF996701.1) and random subtype C sequences (www.ncbi.nlm.nih.gov).

### Cell culture

Human embryonic kidney (HEK) 293T and TZM-bl cells were maintained in Dulbecco modified Eagle high glucose medium (DMEM) (Lonza, Whitehead Scientific). TZM-bl cells were obtained through the NIH AIDS Reagent Program (ARP), Division of AIDS, NIAID from Dr. John C. Kappes, Dr. Xiaoyun Wu and Tranzyme Inc [53]. All cells were grown in a humidified incubator at 37 °C with 5% CO_2_ and growth media were supplemented with 10% fetal bovine serum (FBS) (PAA, Biocom Biotech), 1 U/mL penicillin and 1 µg/mL streptomycin (P/S) (Lonza, Whitehead Scientific).

### Pseudovirus production

Pseudovirus (PSV) was prepared by co-transfection of HEK 293T cells with 2.5 µg of *env* and 5 µg of pSG3ΔEnv plasmids with PEI transfection reagent at a 1:3 DNA: PEI ratio. Culture medium was harvested 48 h later, filtered through a 0.22 µm pore sized filter and FBS was adjusted to 20% and stored at − 70 °C. The concentration of PSV was determined by a chemiluminescent ELISA (Aalto Bio-reagents) and TROPIX^®^ detection system (CDP-Star^®^, Applied Biosystems). PSV titre was determined by infecting TZM-bl cells for 48 h with known p24 concentration and relative luminescence units (RLU) were compared between Env pseudotyped virus and pSG3ΔEnv PSV infected cells (background) and a titre resulting in 50 X RLU above background was used for MDDC stimulation.

### Gp140 production from CHO cells

Full-length *env* was truncated before the transmembrane domain and gp140 was produced as described in Lumngwena et al. [34]. Briefly, Env was expressed in non-adherent CHO cells to maximise expression while preserving N- glycosylation patterns introduced by HEK 293 cells [12]. Culture medium was harvested 72 h post-transfection and gp140 purified by affinity chromatography using *Galanthus nivalis* lectin Sepharose (Vector Labs, Burlingame, CA) diluted fivefold with unliganded Sepharose 4B (GE Healthcare) before dialysis with HEPES, pH 7.4, 150 mM NaCl.

### Generation of MDDC

Peripheral blood mononuclear cells (PBMC) were isolated from buffy coats of healthy blood donors using Ficoll-Hypaque (Sigma-Aldrich). Monocytes isolated from PBMCs by positive selection using CD14+ coated beads (130-050-201 Miltenyi, USA or Biochom Biotech, S.A) were seeded in serum-free medium and allowed to adhere to tissue culture dishes for 2 h at 37 °C, 5% CO_2_. Non-adherent cells were removed and differentiation medium, supplemented with 1000 U/ml GM-CSF (PHC2013, Biosource), and 500 U/ml recombinant human IL-4 (PHC0045, Biosource***)*** was added to the cells and every other day for 6 days.

### Monocyte derived dendritic cell stimulation

Either PSV (50X) or purified gp140 (2 µg/ml) was used to stimulate 2 × 10^6^ cells/mL monocyte derived dendritic cells (MDDC) in 200 µl final volume of culture medium containing 2% AB human serum (H1513, Sigma), 1% P/S (Sigma Aldrich), 1% non-essential amino acids (Gibco-Life technologies, USA), 1% sodium pyruvate (Gibco, USA) and 1% l-glutamine (Sigma Aldrich) in RPMI for 24 h. Following stimulation, the plates were centrifuged at 2000 rpm for 10 min and the culture supernatants collected and stored at − 80℃. Prior to stimulation of MDDCs with PSV, MDDCs were pre-incubated with recombinant DC-SIGN (Donation from Dr Arthos) for 1 h at ambient temperature in the presence of 2 mM Ca^2+^, and then at 37 °C for an additional hour.

### Purification of soluble DC-SIGN

DC-SIGN was cloned into pET22b and expressed in *E coli* as inclusion bodies [54]. Recombinant DC-SIGN was refolded by rapid dilution in renaturation buffer (0.5 M Arginine, 5 mM Cysteamine, 1 mM Cystamine, 0.1 M Tris–HCl, pH 8.0) and purified by ion-exchange (15Q) and Superdex 200 HR size exclusion chromatography [54].

### Luminex multiplex assay for cytokine quantification

The level of cytokines released by MDDCs into the culture supernatants was quantified using Luminex suspension array technology with cytokine-specific antibody-immobilized magnetic beads (Millipore, USA). Biotinylated secondary antibodies and streptavidin–phycoerythrin conjugates were used to determine Mean Fluorescence Intensity (MFI) using a Luminex platform (Bio-rad, USA).

### Phosphorylation of MAP Kinase

MDDCs were rested for 2 h, stimulated with pre-titred PSV or gp140 for 15 min, fixed with 2% paraformaldehyde in PBS, permeabilized and stained with fluorescently conjugated antibodies to ERK and JNK and phosphorylation was detected by flow cytometry. Alternatively, phosphorylation of MAPKs was detected by Western blotting (WB). MDDCs were washed with pre-warmed PBS and lysed (RIPA buffer containing protease and phosphatase inhibitors) after stimulation and 30 μg cell lysate was loaded per well for WB. DC-SIGN binding was inhibited by pre-incubation of MDDCs with anti-DC-SIGN Mab for 2 h at 37 °C (Clone DCN46, ARP 7682).

### Statistical analysis

Cytokine measurements (pg/ml) of at least 3 biological repeats were compared by Mann–Whitney test for non-paired events and Wilcoxon matched test for pairwise comparison of paired events (GraphPad Prism 5.0). A *P* value < 0.05 was considered as statistically significant. P values were adjusted for multiple comparisons by the false discovery rate (FDR) step down procedure. Flow cytometry raw data was analysed by Flowjo 9.6, while the intensity of WB bands was compared by densitometry analysis using the Image lab™ software on a Chemi Doc^Tm^ (Bio-rad) imaging system. Principal component analysis (PCA; in STATA™) was used to reduce the complexity of the dataset and to examine the relationship between PSV and different cytokine functions (chemokine and pro-inflammatory cytokines). Exploratory factor analysis and unsupervised hierarchical clustering (in R) were used to determine the relatedness and uniqueness of the cytokines secreted. The cytokines secreted by MDDCs in response to PSV stimulation were scored in R using factor loading and the degree of variance between cytokines was determined by the eigenvalues. The principal factor method was used and the number of retained factors was determined using the Kaiser’s criterion.

## Data Availability

The datasets generated during and/or analysed during the current study are available in the ZivaHub repository, https://zivahub.uct.ac.za/account/home#/data.

## Abbreviations

FGT: Female genital tract
TF: Transmitter founder
AI: Acute infection
CI: Chronic infection
Env: Envelope
PSV: Pseutotyped Envelops
PBS: Phosphate buffered saline
MAPK: Mitogen activated protein kinases
ERK: Extracellular regulated kinase
JNK: Janus N Kinase
DC: Dendritic cells
MDDC: Monocyte derived dendritic cells
PNG: Potential N-glycosylation Site
PBMC: Peripheral blood mononuclear cells
DC-SIGN: DC specific intracellular adhesion molecule (ICAM) 3 grabbing non-integrin
pDC: Plasmatoid DC
SGA: Single genome amplification
FBS: Fetal bovine serum
HEK: human embryonic kidney cells
SIV: Simian immunodeficiency virus
IR: Immune response
LPS: Lipopolysaccharide
WB: Western blot

## References

[1] Nazli A, Chan O, Dobson-Belaire WN, Ouellet M, Tremblay MJ, Gray-Owen SD, Arsenault AL, Kaushic C (2010). Exposure to HIV-1 directly impairs mucosal epithelial barrier integrity allowing microbial translocation. PLoS Pathog.

[2] Haaland RE, Hawkins PA, Salazar-Gonzalez J, Johnson A, Tichacek A, Karita E, Manigart O, Mulenga J, Keele BF, Shaw GM, Hahn BH, Allen SA, Derdeyn CA, Hunter E (2009). Inflammatory genital infections mitigate a severe genetic bottleneck in heterosexual transmission of subtype A and C HIV-1. PLoS Pathog..

[3] Selhorst P, Masson L, Ismail SD, Samsunder N, Garrett N, Mansoor LE, Karim AQ, Karim ASS, Passmore J-AS, Williamson C (2016). Cervicovaginal inflammation facilitates acquisition of less infectious HIV variants. Infect Dis.

[4] Hladik F, Sakchalathorn P, Ballweber L, Lentz G, Fialkow M, Eschenbach D, McElrath MJ (2007). Initial events in establishing vaginal entry and infection by human immunodeficiency virus type 1. Immunity.

[5] Abrahams M-R, Anderson JA, Giorgi EE, Seoighe C, Mlisana K, Ping L-H, Athreya GS, Treurnicht FK, Keele BF, Wood N, Salazar-Gonzalez JF, Bhattacharya T, Chu H, Hoffman I, Galvin S, Mapanje C, Kazembe P, Thebus R, Fiscus S, Hide W, Cohen MS, Karim SA, Haynes BF, Shaw GM, Hahn BH, Korber BT, Swanstrom R, Williamson C (2009). Quantitating the multiplicity of infection with human immunodeficiency virus type 1 subtype C reveals a non-poisson distribution of transmitted variants. J Virol.

[6] Keele BF, Giorgi EE, Salazar-gonzalez JF, Decker JM, Pham KT, Salazar MG, Sun C, Grayson T, Wang S, Li H, Wei X, Jiang C, Kirchherr JL, Gao F, Anderson JA, Ping L, Swanstrom R, Tomaras GD, Blattner WA, Goepfert PA, Kilby JM, Saag MS, Delwart EL, Busch MP, Cohen MS, Montefiori DC, Haynes BF, Gaschen B, Athreya GS, Lee HY, Wood N, Seoighe C, Perelson AS, Bhattacharya T, Korber BT, Hahn BH, Shaw GM (2008). Identification and characterization of transmitted and early founder virus envelopes in primary HIV-1 infection. Proc Natl Acad Sci..

[7] Wilen CB, Parrish NF, Pfaff JM, Decker JM, Henning EA, Haim H, Petersen JE, Wojcechowskyj JA, Sodroski J, Haynes BF, Montefiori DC, Tilton JC, Shaw GM, Hahn BH, Doms RW (2011). Phenotypic and immunologic comparison of clade B transmitted/founder and chronic HIV-1 envelope glycoproteins. J Virol.

[8] Parrish NF, Gao F, Li H, Giorgi EE, Barbian HJ, Parrish EH, Zajic L, Iyer SS, Decker JM, Kumar A, Hora B, Berg A, Cai F, Hopper J, Denny TN, Ding H, Ochsenbauer C, Kappes JC, Galimidi RP, West AP, Bjorkman PJ, Wilen CB, Doms RW, O’Brien M, Bhardwaj N, Borrow P, Haynes BF, Muldoon M, Theiler JP, Korber B, Shaw GM, Hahn BH (2013). Phenotypic properties of transmitted founder HIV-1. Proc Natl Acad Sci U S A..

[9] Shen R, Raska M, Bimczok D, Novak J, Smith PD (2014). HIV-1 envelope glycan moieties modulate HIV-1 transmission. J Virol.

[10] Raska M, Czernekova L, Moldoveanu Z, Zachova K, Elliott MC, Novak Z, Hall S, Hoelscher M, Maboko L, Brown R, Smith PD, Mestecky J, Novak J (2014). Differential glycosylation of envelope gp120 is associated with differential recognition of HIV-1 by virus-specific antibodies and cell infection. AIDS Res Ther..

[11] Derdeyn CA, Decker JM, Bibollet-Ruche F, Mokili JL, Muldoon M, Denham SA, Heil ML, Kasolo F, Musonda R, Hahn BH, Shaw GM, Korber BT, Allen S, Hunter E (2004). Envelope-constrained neutralization-sensitive HIV-1 after heterosexual transmission. Science..

[12] Go EP, Hewawasam G, Liao H-X, Chen H, Ping L-H, Anderson JA, Hua DC, Haynes BF, Desaire H (2011). Characterization of glycosylation profiles of HIV-1 transmitted/founder envelopes by mass spectrometry. J Virol..

[13] Li Q, Estes JD, Schlievert PM, Duan L, Amanda J, Southern PJ, Reilly CS, Peterson ML, Schultz- N, Brunner KG, Nephew KR, Pambuccian S, Lifson JD, Carlis JV, Haase AT (2009). Glycerol monolaurate prevents mucosal SIV transmission. Nature.

[14] Pollara G, Katz DR, Chain BM (2005). LIGHTing up dendritic cell activation: immune regulation and viral exploitation. J Cell Physiol.

[15] Pollara G, Kwan A, Newton PJ, Handley ME, Chain BM, Katz DR (2005). Dendritic cells in viral pathogenesis: protective or defective?. Int J Exp Pathol.

[16] Fantuzzi L, Purificato C, Donato K, Belardelli F, Gessani S (2004). Human immunodeficiency virus type 1 gp120 induces abnormal maturation and functional alterations of dendritic cells: a novel mechanism for AIDS pathogenesis. J Virol.

[17] Granelli-Piperno A, Golebiowska A, Trumpfheller C, Siegal FP, Steinman RM (2004). HIV-1-infected monocyte-derived dendritic cells do not undergo maturation but can elicit IL-10 production and T cell regulation. Proc Natl Acad Sci U S A..

[18] Geijtenbeek TBH, van Vliet SJ, Koppel EA, Sanchez-Hernandez M, Vandenbroucke-Grauls C, Appelmelk B, van Kooyk Y (2003). Mycobacteria target DC-SIGN to suppress dendritic cell function. J Exp Med.

[19] Borghi P, Fantuzzi L, Varano B, Gessani S, Puddu P, Conti L, Capobianchi MR, Ameglio F, Belardelli F (1995). Induction of interleukin-10 by human immunodeficiency virus type 1 and its gp120 protein in human monocytes/macrophages. Microbiology.

[20] Ji J, Sahu GK, Braciale VL, Cloyd MW (2005). HIV-1 induces IL-10 production in human monocytes via a CD4-independent pathway. Int Immunol.

[21] Buisson S, Benlahrech A, Gazzard B, Gotch F, Kelleher P, Patterson S (2009). Monocyte-derived dendritic cells from HIV type 1-infected individuals show reduced ability to stimulate T cells and have altered production of interleukin (IL)-12 and IL-10. J Infect Dis.

[22] Roberts L, Passmore JS, Williamson C, Bebell LM, Mlisana K, Burgers WA, Van F, Walzl G, Siawaya JFD, Abdool Q, Karim SSA (2010). Plasma cytokine levels during acute HIV-1 infection predict HIV disease progression. Aids..

[23] Stylianou E, Aukrust P, Kvale D, Muller F, Froland SS (1999). IL-10 in HIV infection: increasing serum IL-10 levels with disease progression-down- regulatory effect of potent anti-retroviral therapy. Clin Exp Immunol.

[24] Taoufik Y, Lantz O, Wallon C, Charles a, Dussaix E, Delfraissy JF. Human immunodeficiency virus gp120 inhibits interleukin-12 secretion by human monocytes: an indirect interleukin-10-mediated effect. Blood. 1997;89:2842–8. http://www.ncbi.nlm.nih.gov/pubmed/9108403. 9108403

[25] Borges ÁH, O’Connor JL, Phillips AN, Ronsholt FF, Pett S, Vjecha MJ, French MA, Lundgren JD (2015). Factors associated with plasma IL-6 levels during HIV infection. J Infect Dis..

[26] Borges AH, O’Connor JL, Phillips AN, Rönsholt FF, Pett S, Vjecha MJ, French MA, Lundgren JD (2014). Determinants of IL-6 levels during HIV infection. J Int AIDS Soc..

[27] Breen EC, Rezai AR, Nakajima K, Beall GN, Mitsuyasu RT, Hirano T, Kishimoto T (1990). Infection with HIV is associated with elevated IL - 6 levels and production. J Immunol..

[28] Chehimi J, Stuart E, Frank I, D’Andrea A, Ma X, MacGregor R, Sennelier J, Trinchieri G (1994). Impaired interleukin 12 production in human immunodeficiency virus- infected patients. J Exp Med.

[29] Daftarian MP, Diaz-Mitoma F, Creery WD, Cameron W, Kumar a. Dysregulated production of interleukin-10 (IL-10) and IL-12 by peripheral blood lymphocytes from human immunodeficiency virus-infected individuals is associated with altered proliferative responses to recall antigens. Clin Diagn Lab Immunol. 1995;2:712–8. http://www.pubmedcentral.nih.gov/articlerender.fcgi?artid=170227&tool=pmcentrez&rendertype=abstract. 10.1128/cdli.2.6.712-718.1995 PMC170227 8574836

[30] Jennes W, Vereecken C, Fransen K, De Roo A, Kestens L. Disturbed secretory capacity for macrophage inflammatory protein (MIP)-1α and MIP-1β in progressive HIV infection. AIDS Res Hum Retroviruses. Mary Ann Liebert, Inc., publishers; 2004;20:1087–1091. 10.1089/aid.2004.20.1087. 10.1089/aid.2004.20.1087 15585099

[31] Matsumoto T, Miike T, Nelson RP, Trudeau WL, Lockey RF, Yodoi J (1993). Elevated serum levels of IL-8 in patients with HIV infection. Clin Exp Immunol.

[32] Marshall JD, Chehimi J, Gri G, Kostman JR, Montaner LJ, Trinchieri G. The interleukin-12-mediated pathway of immune events is dysfunctional in human immunodeficiency virus-infected individuals. Blood. 1999;94:1003–11. http://www.ncbi.nlm.nih.gov/pubmed/10419892. 10419892

[33] Bebell LM, Passmore J, Williamson C, Mlisana K, Iriogbe I, Van Loggerenberg F, Karim QA, Karim SA (2008). Relationship between levels of inflammatory cytokines in the genital tract and CD4+ cell counts in women with acute HIV-1 infection. J Infect Dis.

[34] Lumngwena EN, Abrahams B, Shuping L, Cicala C, Arthos J, Id ZW (2020). Selective transmission of some HIV-1 subtype C variants might depend on Envelope stimulating dendritic cells to secrete IL-10. PLoS Negl Trop Dis..

[35] Hodges A, Sharrocks K, Edelmann M, Baban D, Moris A, Schwartz O, Drakesmith H, Davies K, Kessler B, McMichael A, Simmons A (2007). Activation of the lectin DC-SIGN induces an immature dendritic cell phenotype triggering Rho-GTPase activity required for HIV-1 replication. Nat Immunol.

[36] Gringhuis SI, Van Der Vlist M, Van Den Berg LM, Den Dunnen J, Litjens M, Geijtenbeek TBH (2010). HIV-1 exploits innate signaling by TLR8 and DC-SIGN for productive infection of dendritic cells. Nat Immunol.

[37] Shan M, Klasse PJ, Banerjee K, Dey AK, Iyer SPN, Dionisio R, Charles D, Campbell-Gardener L, Olson WC, Sanders RW, Moore JP (2007). HIV-1 gp120 mannoses induce immunosuppressive responses from dendritic cells. PLoS Pathog.

[38] Geijtenbeek TBH, Gringhuis SI (2009). Signalling through C-type lectin receptors: shaping immune responses. Nat Rev Immunol.

[39] Wilflingseder D, Mullauer B, Schramek H, Banki Z, Pruenster M, Dierich MP, Stoiber H (2004). HIV-1-induced migration of monocyte-derived dendritic cells is associated with differential activation of MAPK pathways. J Immunol..

[40] Banchereau J, Steinman R (1998). Dendritic cells and the control of immunity. Nature.

[41] Steinman R (2006). Hemmi H.

[42] MacCallum RC, Widaman KF, Zhang SB, Hong SH (1999). Sample size in factor analysis. Psychol Methods.

[43] Rescigno M, Martino M, Sutherland CL, Gold MR, Ricciardi-Castagnoli P (1998). Dendritic cell survival and maturation are regulated by different signaling pathways. J Exp Med.

[44] Arnold KB, Burgener A, Birse K, Romas L, Dunphy LJ, Shahabi K, Abou M, Westmacott GR, McCorrister S, Kwatampora J, Nyanga B, Kimani J, Masson L, Liebenberg LJ, Karim ASS, Passmore J-AS, Lauffenburger DA, Kaul R, McKinnon LR (2015). Increased levels of inflammatory cytokines in the female reproductive tract are associated with altered expression of proteases, mucosal barrier proteins, and an influx of HIV-susceptible target cells. Mucosal Immunol.

[45] Anahtar MN, Byrne EH, Doherty KE, Bowman BA, Yamamoto S, Soumillon M, Padavattan N, Ismail N, Moodley A, Sabatini ME, Ghebremichael MS, Nusbaum C, Huttenhower C, Virgin HW, Ndung T, Dong KL, Walker BD, Raina N (2015). Cervicovaginal bacterial are major modulator of host inflammatory responses in the female genital tract. Immunity.

[46] Masson L, Passmore J-AS, Liebenberg LJ, Werner L, Baxter C, Arnold KB, Williamson C, Little F, Mansoor LE, Naranbhai V, Lauffenburger DA, Ronacher K, Walzl G, Garrett NJ, Williams BL, Couto-Rodriguez M, Hornig M, Lipkin WI, Grobler A, Karim AQ, Karim ASS (2015). Genital inflammation and the risk of HIV acquisition in women. Clin Infect Dis.

[47] Stacey AR, Norris PJ, Qin L, Haygreen EA, Taylor E, Heitman J, Lebedeva M, DeCamp A, Li D, Grove D, Self SG, Borrow P (2009). Induction of a striking systemic cytokine cascade prior to peak viremia in acute human immunodeficiency virus type 1 infection, in contrast to more modest and delayed responses in acute hepatitis B and C virus infections. J Virol.

[48] Gray ES, Moore PL, Choge IA, Decker JM, Li H, Leseka N, Treurnicht F, Mlisana K, Shaw GM, Karim SSA, Williamson C, Morris L, Team S (2007). Neutralizing antibody responses in acute human immunodeficiency virus Type 1 subtype C infection. J Virol.

[49] Gringhuis SI, den Dunnen J, Litjens M, van het Hof B, van Kooyk Y, Geijtenbeek TBH (2007). C-type LECTIN DC-SIGN modulates toll-like receptor signaling via Raf-1 kinase-dependent acetylation of transcription factor NF-κB. Immunity.

[50] Feinberg H, Castelli R, Drickamer K, Seeberger PH, Weis WI (2007). Multiple modes of binding enhance the affinity of DC-SIGN for high mannose N-linked glycans found on viral glycoproteins. J Biol Chem.

[51] Drickamer K, Taylor ME (2015). Recent insights into structures and functions of C-type lectins in the immune system. Curr Opin Struct Biol. Elsevier Ltd.

[52] Roberts L, Passmore JAS, Mlisana K, Williamson C, Little F, Bebell LM, Walzl G, Abrahams MR, Woodman Z, Karim QA, Karim SSA (2012). Genital tract inflammation during early HIV-1 infection predicts higher plasma viral load set point in women. J Infect Dis.

[53] Platt EJ, Wehrly K, Kuhmann SE, Chesebro B, Kabat D (1998). Effects of CCR5 and CD4 cell surface concentrations on infections by macrophagetropic isolates of human immunodeficiency virus type 1. J Virol.

[54] Snyder GA, Ford J, Torabi-Parizi P, Arthos JA, Schuck P, Colonna M, Sun PD (2005). Characterization of DC-SIGN/R interaction with human immunodeficiency virus type 1 gp120 and ICAM molecules favors the receptor’s role as an antigen-capturing rather than an adhesion receptor. J Virol.

